# RB1 expression and HR proficiency define a poor prognosis subtype of high grade serous ovarian cancer

**DOI:** 10.1038/s41598-025-15156-9

**Published:** 2025-08-12

**Authors:** Kyle C. Strickland, Zachary D. Wallen, Heidi C. Ko, Michelle F. Green, Alicia Dillard, Sarabjot Pabla, Stephanie Hastings, Alison Roos, Taylor J. Jensen, Marcia Eisenberg, Brian J. Caveney, Shakti Ramkissoon, Eric A. Severson, Rebecca A. Previs

**Affiliations:** 1 Labcorp, 10 Moore Dr, Durham, NC 27703 USA; 2 Department of Pathology, Duke University Medical Center, Duke Cancer Institute, Durham, NC 27710 USA; 3 Thermo Fisher Scientific, Carlsbad, CA 92008 USA; 4 Labcorp, Burlington, NC 27215 USA; 5 Department of Pathology, Wake Forest Comprehensive Cancer Center, Wake Forest School of Medicine, Winston-Salem, NC 27109 USA; 6 Department of Obstetrics & Gynecology, Division of Gynecologic Oncology, Duke University Medical Center, Duke Cancer Institute, Durham, NC 27710 USA

**Keywords:** High grade serous ovarian carcinoma, Papillary serous adenocarcinoma, Ovarian cancer, Gynecologic oncology, Homologous recombination deficiency, RB1, Ovarian cancer, Cancer genomics

## Abstract

High-grade serous ovarian carcinoma (HGSOC) is a molecularly heterogeneous and lethal malignancy, with late-stage diagnosis contributing to high risk of recurrence and poor clinical outcomes. Although homologous recombination (HR) deficiency and retinoblastoma gene (*RB1*) expression have been implicated in prognosis, their combined role in shaping tumor biology and survival outcomes is not well defined. To investigate the relationship between HR status and RB1 expression and explore their potential as a combined prognostic marker, we analyzed data from two cohorts: (1) 272 HGSOC cases from The Cancer Genome Atlas (TCGA) with *RB1* mRNA expression data and HR status previously annotated by Takaya et al. (HR-deficient, HRD; HR-proficient, HRP), and (2) 226 clinical HGSOC cases profiled by comprehensive genomic and immune profiling (CGIP) at OmniSeq, categorized as either HR-intact (HRi) or harboring *BRCA1/2* alterations (BRCAa). Cases were additionally stratified according to RB1 mRNA expression level as RB1-high (> 25th percentile; RBH) or RB1-low (≤ 25th percentile; RBL). HRP-RBH tumors (*n* = 120, 44.1%) were associated with significantly worse overall survival (OS) and progression free survival (PFS) compared to all other subgroups. Survival metrics were evaluated from the TCGA cohort and demonstrated that median OS for HRP-RBH was 35.9 mo, shorter than HRP-RBL (52.0 mo), HRD-RBL (57.1 mo), and HRD-RBH subgroups (53.3 mo; all *p* < 0.0001). PFS demonstrated a similar trend (15.1 mo vs. 20.6, 20.2 and 20.4 mo, respectively, *p* = 0.0021). HRP-RBH tumors also showed higher aneuploidy scores (median 18 vs. ≤ 10.5 in other subgroups, all *p* < 0.01). From the OmniSeq cohort, HRi-RBH tumors exhibited a distinct immune gene signature, including elevated mRNA expression of 213 differentially expressed genes and enrichment of pathways such as EMT, PI3K/AKT signaling, and interleukin signaling. Overall, this study suggests that molecular subtyping of HGSOC based on HR status and *RB1* expression may provide valuable prognostic insight. HRP tumors with high *RB1* expression represent a high-risk subgroup with a distinct molecular profile and poor clinical outcomes, underscoring the need for novel therapeutic strategies targeting this aggressive subset. These findings provide a foundation for future studies aimed at developing biomarkers and treatments tailored to this challenging subset of HGSOC patients.

**Supplementary Information:**

The online version contains supplementary material available at 10.1038/s41598-025-15156-9.

## Introduction

High-grade serous ovarian carcinoma (HGSOC) represents the most lethal subtype of ovarian cancer, characterized by its late detection and poor prognosis^1,2^. Despite advancements in treatment, the five-year survival rate remains extremely low^3^, emphasizing the critical need for improved molecular insights and therapeutic strategies. HGSOC is marked by significant molecular heterogeneity, with diverse genetic and epigenetic landscapes influencing tumor behavior and response to treatment^4^. Approximately 50% of HGSOC have defects in homologous recombination (HR), a key cellular mechanism for repairing double-stranded DNA breaks^5^. Tumors deficient in HR (HRD) are particularly susceptible to specific chemotherapies, such as those inducing DNA damage (such as platinum-based chemotherapies) and those that inhibit the alternative non-homologous end joining pathway of repairing double-stranded DNA breaks (i.e., PARP inhibitors)^6–8^.

Approximately 40% of HGSOCs have alterations of genes regulating the G1 to S cell cycle transition, including participants in the retinoblastoma (*RB1*) pathway such as *CDKN2A*, *CCND1*, *CDK4*, and *CDK6*^9^. *RB1* encodes a fundamental tumor suppressing protein that regulates the cell cycle by directly binding to and suppressing activity of transcription factors E2F1/2/3 and thus controlling the transition from the G1 to the S phase^10,11^. Alterations in *RB1* and its cellular partners disrupt this control and are implicated in the pathogenesis of various cancers, including HGSOC^12–15^. Studies of HGSOC have demonstrated that loss of RB1 protein expression is associated with improved prognosis^16^, particularly when seen in conjunction with HRD^17–19^, but RB1 expression status is not incorporated into any clinical diagnostic, treatment, or prognostic paradigms at present.

The molecular mechanisms underlying these associations remain uncertain. Increased understanding of the clinical course and molecular characteristics of HGSOC with respect to HR and RB1 status may enable prognostic tools and potential therapies. The goal of the current study is to explore the relationship between HR status and RB1 mRNA expression in HGSOC, focusing on their combined impact on clinical outcomes, genomic features, and gene expression changes. We hypothesize that RB1 mRNA expression, as a surrogate marker of RB1 protein expression, is associated with survival outcomes and immune-related gene expression changes depending on the HR status of HGSOC tumors. The current study validates the prognostic value of RB1 within the context of HR proficient and deficient HGSOC and provides insight into the biological consequences of these combined alterations.

## Materials and methods

### Statistical analysis of cases from the HGSOC TCGA database

Mutation, mRNA expression, copy number alteration (CNA), and protein quantification data were obtained from the Ovarian Serous Cystadenocarcinoma TCGA PanCancer database found on cBioportal^4,20–22^. Mutation data was processed to filter out silent mutations and create a binary mutation matrix. RB1 CNA status was evaluated by creating contingency tables with separate data frames for cases with RB1 deep deletion, hemi-deletion, and no change/gain. Aneuploidy score was defined as the total number of altered chromosome arms, ranging from 0 (no altered arms) to 39 (alterations in all chromosome arms). As an absolute inclusion criterion, our study was restricted to HGSOC cases from TCGA that were evaluated by Takaya et al.^23^from which both HRD status and HRD scores were abstracted; inclusion in our study required availability of both metrics. Survival analyses were performed by grouping according to the scoring system used by Takaya et al.: (1) HR status score, with ≥ 63 considered HR-deficient (HRD) and < 63 considered HR-proficient (HRP), and (2) RNA-seq RB1 mRNA expression quartile, with ≤ 25th percentile for all TCGA cases considered RB-low (RBL) and > 25th percentile considered RB1-high (RBH). Protein quantification data were acquired from the cBioPortal Firehose generated by the Clinical Proteomic Tumor Analysis Consortium (CPTAC) via mass spectrometry, and protein values represent normalized, relative abundance levels on a log2 scale. Statistical analyses were performed using R statistical software (version 4.2.0)^24^. Kaplan-Meier curves were fitted using the survival R package and plotted using ggplot2 and survminer^24–26^. Continuous variables were compared using the Mann-Whitney test. Statistical comparisons of RB1 protein levels were performed using the Wilcoxon rank-sum test for non-parametric pairwise analysis.

### Comprehensive genomic and immune gene expression profiling

This study was approved by the Western Copernicus Group Institutional Review Board (WCG IRB, protocol #1340120). All methods were carried out in accordance with relevant guidelines and regulations, including those governing the ethical use of human-derived data. The requirement for informed consent was waived by the WCG IRB under 45 CFR 164.512, as the study involved retrospective analysis of de-identified clinical data. We queried our institutional database for cases of ovarian serous tumors that underwent comprehensive genomic and immune profiling (CGIP) at our laboratory (OmniSeq^®^, Labcorp Oncology) from June 2016 to October 2023. Cases were included in the cohort if they met the following criteria: (1) pathology reports from referring institutions contained a diagnosis of ovarian serous carcinoma, (2) tissue slides were reviewed by a board-certified pathologist to confirm concordance with serous morphology, and (3) tumors harbored at least one pathogenic somatic alteration in the *TP53* gene, to enrich for high grade cases. Cases were subjected to testing using one of three assays: OmniSeq Comprehensive (OC), OmniSeq Advance (OA), or OmniSeq INSIGHT (OI) (Labcorp Oncology, Durham, NC). Each of these tests uses next-generation sequencing of DNA and/or RNA to profile solid tumors. OC (now retired) was an amplicon-based molecular panel that tested for mutations in 144 genes, fusions in 23 genes, and copy number changes. OC was later combined with a 54-gene mRNA expression profiling test, OmniSeq Immune Report Card (IRC), to define the OA test, which assessed both genomic alterations and mRNA gene expression^27^. Of note, OC/OA interrogated only a select number of HR genes including *ATM*,* BAP1*,* BRCA1*,* BRCA2*, and *PTEN*. The third test, OI, utilized extracted DNA and RNA for genomic and immune profiling and includes DNA sequencing performed to evaluate 523 genes for SNVs, insertions, deletions, 59 genes for copy number amplifications, and 4 genes for copy number loss^28^. Tumor mutational burden was determined from small variant output, and microsatellite instability was assessed using up to 130 specific microsatellite sites. RNA sequencing was performed to evaluate 55 genes for fusions and 2 genes for splice variants, and immune gene expression profiling was achieved through RNA-sequencing of 395 immune-related transcripts. OI testing also includes PD-L1 immunohistochemistry, which utilizes 22C3 antibody for ovarian tumors and was scored using the tumor proportion score (TPS) method. We evaluated the following gene expression signatures as previously described: tumor immunogenicity score (TIGS), cellular proliferation (CP), and cancer testis antigen burden (CTAB)^29,30^.

Cases were included in the study provided the tumor harbored at least one pathogenic mutation in *TP53* (as defined by ClinVar) and were stratified based on the presence or absence of genomic alterations in HR pathway genes. Cases with any pathogenic alteration in either *BRCA1* or *BRCA2* were classified as *BRCA*-altered (BRCAa). Cases with normal *BRCA1* and *BRCA2* sequences but harboring pathogenic alterations in other HR genes (*BRCA1*,* BRCA2*,* ATM*,* ATR*,* BARD1*,* BLM*,* BRIP1*,* CDK12*,* CHEK1*,* CHEK2*,* FANCA*,* FANCC*,* FANCD2*,* FANCE*,* FANCF*,* FANCI*,* FANCL*,* FANCM*,* MRE11*,* NBN*,* PALB2*,* RAD50*,* RAD51*,* RAD51B*,* RAD51C*,* RAD51D*,* RAD52*,* RAD54L*, and *RPA1*) were classified as non-BRCA-HR-altered (NBHRa), per the ARIEL3 study and other reports^23,31,32^. Cases with no pathogenic alterations in any HR genes were classified as HR-intact (HRi). Of note, this system of stratification is distinct from that used to evaluate the TCGA dataset, which utilized an HRD score to determine HR status.

*Statistical analysis of the OmniSeq HGSOC cohort*.

We compared TMB, PD-L1 IHC, nRPM (normalized reads per million mapped reads) values, and gene expression signature scores between cohorts. Differential gene expression between cohorts was assessed for all 395 immune-related transcripts and selected transcripts of interest, including *RB1*, *BRCA1*, *BRCA2*, *CD3* (calculated as the average of *CD3D*, *CD3E*, and *CD3G*), *CD68*, *CD20* (calculated as the average of *CD20A* and *CD20B*), *PTEN*, and *MK67*. Continuous and categorical variables were evaluated using linear or Firth’s penalized logistic regression adjusting for the type of assay used to perform genomic and immune profiling (OC, OA, or OI). Continuous variables with positive skewness (TMB, nRPM values, CTAB signature scores) were given a pseudo-count of 1 to avoid 0 values and log transformed before testing. Estimated fold changes for gene expression changes between cohorts were calculated by taking the exponent of the regression coefficients from linear and Firth’s penalized regression. Multiple testing correction of P-values resulting from individual immune gene expression analysis was performed using the Benjamini-Hochberg false discovery rate (FDR) method. A significance threshold of < 0.05 and < 0.25 was used for uncorrected P-values and multiple testing corrected P-values, respectively. Genes in which expression was found to be significantly different between groups (FDR < 0.25) are referred to as differentially expressed genes (DEGs) between those groups. Of note, the < 0.25 threshold is a commonly used threshold for *P*-values that have been multiple testing corrected using the FDR procedure and when testing large numbers of features such as in pathway enrichment^33,34^.

### Pathway analysis of differentially expressed genes using the PANTHER classification system

Lists of DEGs between BRCAa, HRi, and NBHRa groups of the OmniSeq cohort were submitted for pathway analysis utilizing the PANTHER (Protein ANalysis THrough Evolutionary Relationships) Classification System (https://pantherdb.org) to identify Reactome pathways related to DEGs based on a reference list of all genes from the *Homo sapiens* whole genome^35,36^. Reactome (Reactome version 86, released on 09-07-2023) is an open-source relational database of signaling and metabolic molecules, organized into biological pathways and processes, with reactions serving as its core unit^37^. Fisher’s Exact test was applied to assess statistical significance of pathway enrichment, and FDR was calculated to correct for multiple testing. Pathways with a corrected p-value (FDR) < 0.05 were identified as DEG-related.

## Results

### Demographics, molecular characteristics, and survival analysis of the ovarian cancer cases from the TCGA PanCancer atlas stratified by HR and RB1 expression status

The Ovarian Serous Cystadenocarcinoma TCGA PanCancer Atlas dataset contained mutation-level data for 409 cases, which included 373 (91.2%) tumors harboring pathogenic alterations in *TP53*. For subgroup and survival analyses, we abstracted data for TCGA cases with HR scores reported by Takaya et al. that also had mRNA expression data available^23^, producing an experimental cohort of 272 cases consisting of 150 (55.1%) HRP and 122 (44.9%) HRD tumors (Supplemental Table 1). Patients in the HRP cohort were older (median age 60 vs. 55.5 years, *p* = 0.0002). The median HRD score was 48 for HRP and 77 for the HRD tumors, with median loss of heterozygosity (LOH) scores of 8 and 18, median telomeric-allelic imbalance (TAI) scores of 21 and 29, and median large-scale state transition (LST) scores of 17.5 and 30, respectively. HRP tumors harbored lower TMB (median 1.5 vs. 2.2 mutations/Megabase, *p* = 0.0019) but demonstrated higher aneuploidy scores (median 17 vs. 10, *p* < 0.0001). HRP tumors demonstrated significantly increased RB1 mRNA expression levels, with a median of 983 normalized transcripts per million (nTPM) compared to 884 nTPM in HRD tumors (*p* < 0.0001). Ki-67 mRNA expression was similar for both HRP and HRD groups (median 2191 vs. 2253, respectively, *p* > 0.05).

We further stratified the HRP and HRD cohorts by RB1 mRNA expression level, with ≥ 701 nTPM (the 25th percentile) as the cut-off for classification into either RBH or RBL groups (Supplemental Table 1). Of HRP tumors (*n* = 150), 120 (80%) were RBH and 30 (20%) were RBL. Of HRD tumors (*n* = 122), 84 (68.9%) were RBH and 38 (31.1%) were RBL. Based on this simple classification, we observed an increased proportion of RBH tumors among the HRP tumors (*p* = 0.048, Fisher’s exact test). Patients in the HRP-RBH group were older than those in either the HRD-RBH (median age 60 vs. 55 years, *p* = 0.0006) or HRD-RBL (median age 60 vs. 56 years, *p* = 0.039). HRP-RBH and HRP-RBL groups were comprised of patients of similar age (60 vs. 59.5 years, respectively, *p* = 0.78). We did not observe a statistically significant difference in ages of patients in the HRP-RBL group versus either the HRD-RBH (59.5 vs. 55 years, *p* > 0.05) or the HRD-RBL (59.5 vs. 56 years, *p* > 0.05) groups.

To confirm that low RB1 mRNA was associated with low protein expression, we evaluated TCGA cases that had both RB1 protein quantification and mRNA expression data. We identified 73 cases from the TCGA cohort with these values, which included 12 (16%) HRP-RBL, 29 (40%) HRP-RBH, 5 (7%) HRD-RBL, and 27 (37%) HRD-RBH samples. Relative RB1 protein expression levels were significantly lower in RBL tumors compared to RBH tumors (median − 0.59 vs. 0.20, *p* = 3.7 × 10⁻⁶). When stratified by HRD status, RB1 protein was significantly reduced in RBL vs. RBH tumors among HRP cases (median − 0.40 vs. 0.23, *p* = 7.4 × 10⁻⁶). A similar trend was observed in HRD tumors (median − 0.85 vs. 0.088), though it did not reach statistical significance (*p* = 0.062). No significant difference in RB1 protein levels was found when comparing all HRP vs. HRD tumors irrespective of RB1 expression (median 0.072 vs. 0.026, *p* = 0.99). Cross-comparisons of RB1 expression and HRD status showed that HRD/RBL tumors had significantly lower RB1 protein than HRP/RBH tumors (median − 0.85 vs. 0.23, *p* = 0.020). Conversely, HRD/RBH tumors had significantly higher RB1 protein than HRP/RBL tumors (median 0.088 vs. − 0.40, *p* = 1.2 × 10⁻⁴).

**Fig. 1:**
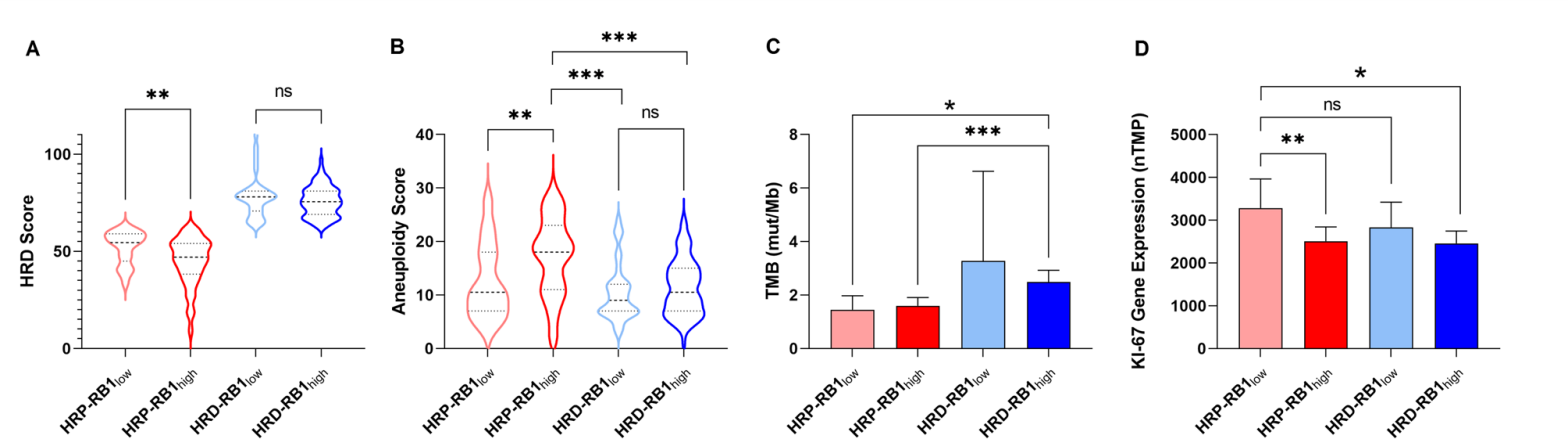
Molecular characteristics of cases from the Ovarian Serous Cystadenocarcinoma TCGA dataset. We compared **(A)** HRD scores, **(B)** aneuploidy scores, **(C)** tumor mutational burden, and **(D)** Ki-67 gene expression for each molecular subgroup we identified from the ovarian TCGA data. ns *P* > 0.05, * *P* ≤ 0.05, ** *P* ≤ 0.01, *** *P* ≤ 0.001.

HRD, LST, LOH, and TAI scores reported by Takaya et al. 2020 were compared within HRP and HRD groups to evaluate potential associations with low or high RB1 expression. Within the HRP cohort, RBH tumors demonstrated lower HRD scores than RBL tumors (47 vs. 54.5, *p* = 0.0016), with significantly lower LOH scores (7 vs. 10, *p* = 0.0300) (Fig. 1A), significantly lower LST scores (17 vs. 22, *p* = 0.0023), and no difference in TAI scores (21 vs. 21, *p* > 0.05). Comparing HRD-RBH and HRD-RBL tumors, we did not observe statistically significant differences in HRD (75.5 vs. 78, *p* > 0.05), LOH (18 vs. 19; *p* = 0.0624), LST (30 vs. 29.5, *p* > 0.05) or TAI (29 vs. 28, *p* > 0.05) scores, respectively. Median aneuploidy scores were markedly higher in the HRP-RBH group compared to the other cohorts: HRP-RBL (18 vs. 10.5, *p* = 0.0013), HRD-RBL (18 vs. 9, *p* < 0.0001), and HRD-RBH (18 vs. 10.5, *p* < 0.0001) (Fig. 1B). TMB varied across groups, with the lowest observed in the HRP-RBL group (1.3 mut/Mb) and the highest in the HRD-RBH group (2.3 mut/Mb). The differences in TMB reached statistical significance between HRP-RBH vs. HRD-RBH groups (1.5 vs. 2.3 mut/Mb, respectively; *p* = 0.0003) and HRP-RBL vs. HRD-RBH groups (1.3 vs. 2.3 mut/Mb, respectively; *p* = 0.0161) (Fig. 1C). There was no difference in RB1 mRNA expression scores within HRP and HRD groups after stratifying samples as RBL or RBH: HRP-RBH vs. HRD-RBH (1100 vs. 1070 nTPM, *p* > 0.05) and HRP-RBL vs. HRD-RBL (523 vs. 479 nTPM, *p* > 0.05). Ki-67 mRNA expression levels were highest in the HRP-RBL group, which was statistically higher than that of the HRP-RBH (3135 vs. 2082 nTPM, *p* = 0.0078) and the HRD-RBH (3135 vs. 2210, *p* = 0.028) (Fig. 1D). There was no statistically significant difference in Ki-67 mRNA expression between HRP-RBL and HRD-RBL groups (3135 vs. 2277, *p* > 0.05).

**Fig. 2:**
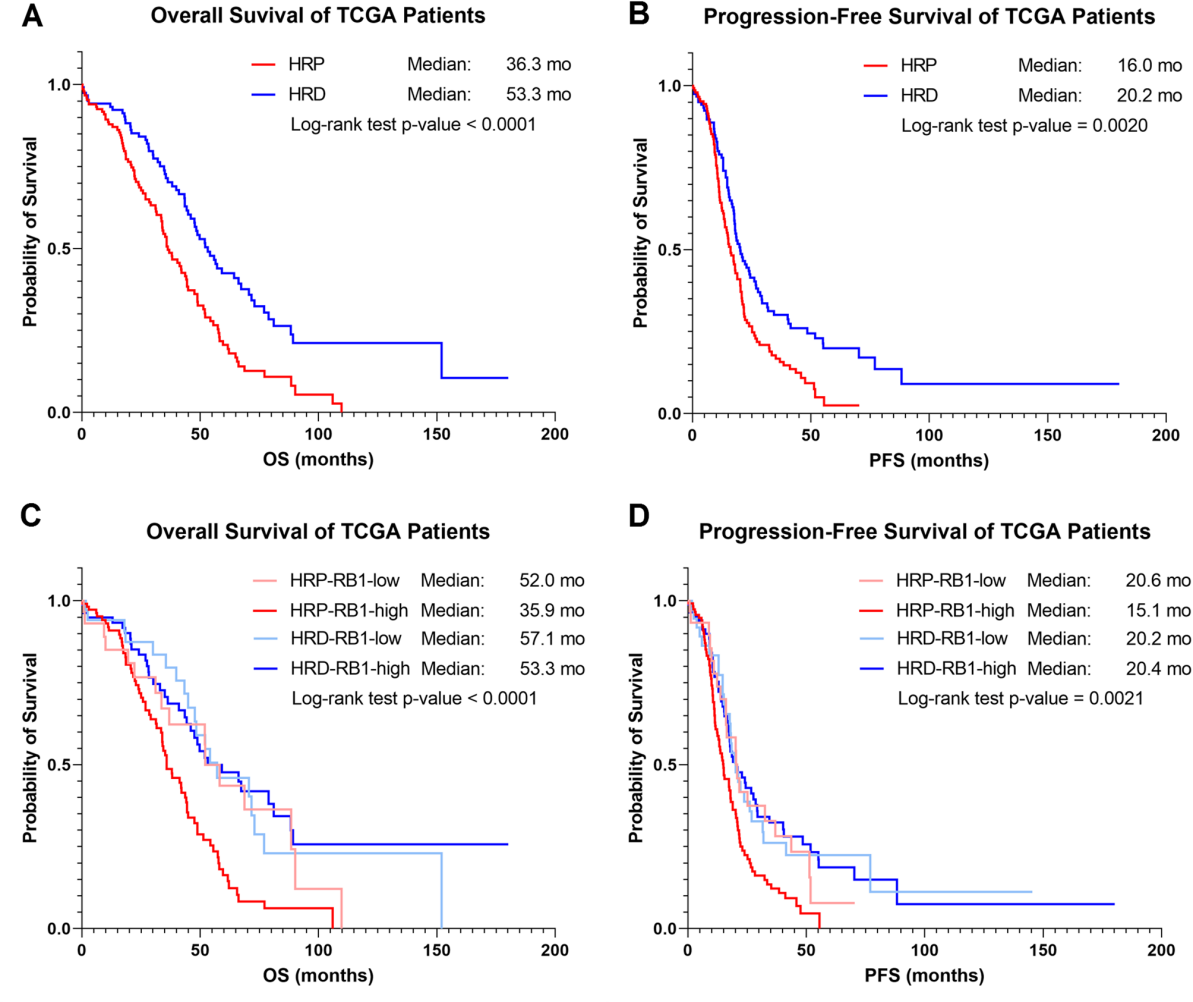
Survival of HGSOC TCGA patients stratified by HR status and *RB1* expression status. TCGA patients were stratified according to the HR status of their tumor as published by Takaya et al. 2020, and Kaplain Meier curves were generated comparing **(A)** overall survival and **(B)** progression-free survival between HRP and HRD cohorts. Patients were further stratified based on the level of RB1 mRNA expression, and Kaplan Meier curves were generated comparing **(C)** overall survival and **(D)** progression-free survival for the various subgroups.

Patients in the HRP cohort demonstrated significantly shorter OS (36.3 vs. 53.3 months, *p* < 0.0001) and PFS (16.0 vs. 20.2 months, *p* = 0.0020) than those with HRD tumors (Fig. 2A-B). After stratifying patients according to RB1 mRNA expression levels, survival outcomes for HRP-RBH patients were shorter compared to all other groups. Among HRP tumors, those with RBH had lower median OS (35.9 vs. 52.0 months, *p* = 0.015) and PFS (15.1 vs. 20.6 months, *p* = 0.031) (Fig. 2C-D). Among RBH expressing tumors, HRP patients had markedly lower survival metrics compared to HRD-RBH tumors, including lower median OS (35.9 vs. 57.1 months, *p* < 0.0001), and PFS (15.1 vs. 20.2 months, *p* = 0.0007). Similarly, compared to the HRD-RBL group, HRP-RBH tumors were associated with decreased OS (35.9 vs. 57.1 months, *p* = 0.0017) and PFS (15.1 vs. 20.2 months, *p* = 0.013). In contrast to HRP-RBH tumors, we did not observe statistically significant differences in survival metrics between HRP-RBL tumors and either HRD-RBH and HRD-RBL groups. Among the RBL groups, HRP-RBL patients demonstrated a shorter median OS (52.0 vs. 57.1 months, *p* > 0.05) and longer PFS (20.6 vs. 20.2 months, *p* > 0.05) than HRD-RBL patients. Similar results were obtained when compared to HRD-RBH patients, with HRP-RBH patients having a shorter median OS (52.0 vs. 53.3 months, *p* > 0.05) and longer PFS (20.6 vs. 20.4 months, *p* > 0.05). Additionally, we did not observe a statistically significant difference in survival between the two groups of HRD patients; HRD-RBH patients demonstrated lower median OS (53.3 vs. 57.1 months, *p* > 0.05) and comparable PFS (20.4 vs. 20.2 months, *p* > 0.05).

We identified 233 cases from the TCGA dataset that contained gene-level copy number information, in addition to mRNA expression data and HR evaluation by Takaya et al.^23^ The majority of HGSOC contained hemizygous deletion of the *RB1* gene (56.7%), 25.3% of cases were copy number neutral, 8.2% of cases had homozygous deletion of *RB1*, and 9.9% had amplification of the *RB1* gene (Supplemental Table 2). RB1 mRNA expression was tightly correlated with *RB1* copy number status: cases with homozygous deletion of *RB1* demonstrated the lowest gene expression (median 486 nTPM), cases with hemizygous deletion of *RB1* had lower expression than *RB1* copy number neutral cases (median 865 vs. 1135 nTPM), and cases with amplification demonstrated the highest gene expression (median 1525 nTPM). Only 2 of 19 (10.5%) cases with homozygous deletion demonstrated high levels of RB1 gene expression (TCGA-24-2262 and TCGA-61-2109). Among cases with hemizygous deletion of *RB1*, 95 of 132 (72.0%) were RBH, and two cases with hemizygous deletion of *RB1* harbored single nucleotide variants in *RB1*: (1) TCGA-24-1469 harbored R763T and had an RB1 gene expression of 1644 nTPM and (2) TCGA-29-1703 harbored a hotspot splice site mutation (X405) and had an RB1 gene expression of 717 nTPM. Just 4 of 59 (6.8%) of *RB1* copy number neutral cases were RBL, and 3 of these 4 (75%) had detectable, pathogenic SNVs in *RB1*: (1) TCGA-61-1741 harbored a splice site alteration (X474) and had an RB1 gene expression of 319 nTPM, (2) TCGA-24-1849 harbored a frameshift mutation (F526Lfs*) and had an RB1 gene expression of 612 nTPM, and (3) TCGA-24-1463 harbored a frameshift mutation (C553Wfs*) and had an RB1 gene expression of 480 nTPM). None of the cases with amplification of *RB1* were RBL or harbored SNVs in *RB1*.

### HR gene status is associated with divergent immune gene expression profiles

**Fig. 3:**
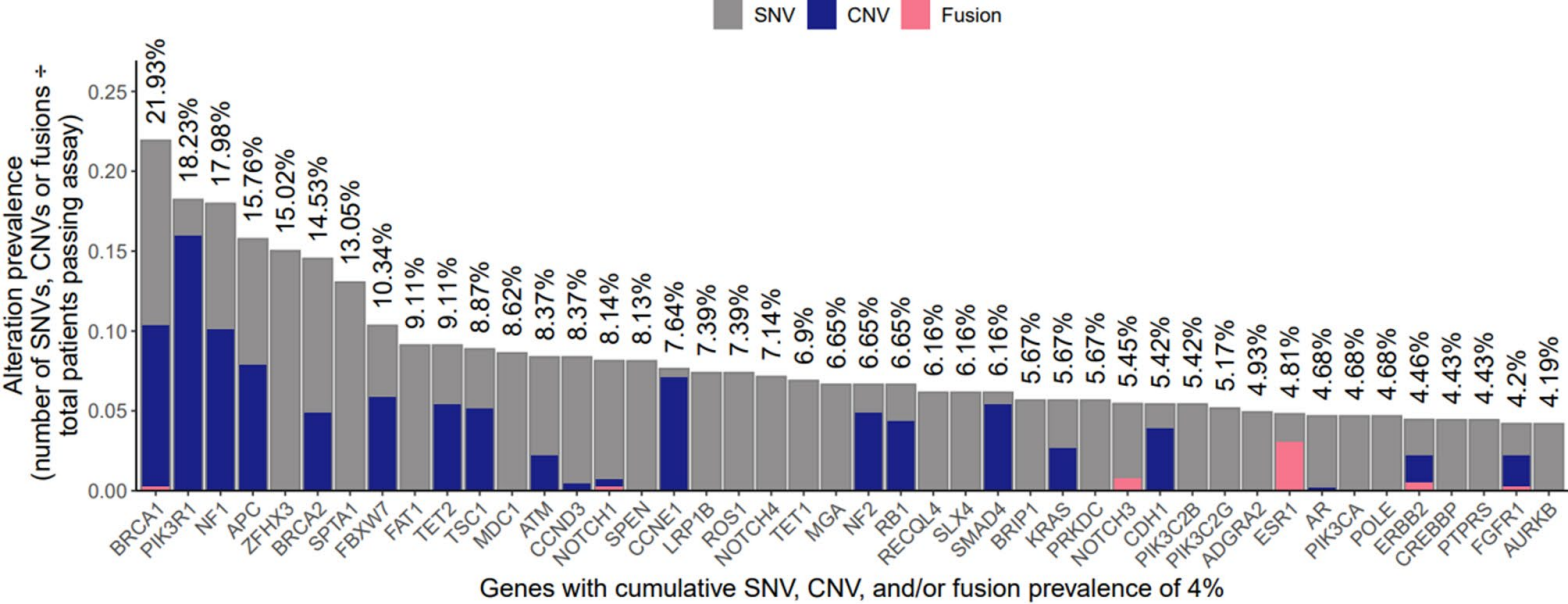
Genomic landscape of cases from the OmniSeq HGSOC cohort. The histogram below shows the prevalence and type of alterations for the most commonly altered genes in the OmniSeq HGSOC cohort. Of note, 100% of the tumors harbored pathogenic alterations in *TP53* (not shown).

We next investigated associations between *RB1* and HR status in a separate cohort of HGOSC that were processed for clinical CGIP by our laboratory. We identified 406 ovarian high grade serous tumors from 249 provider facilities across the United States that had CGIP performed by OmniSeq and met inclusion criteria, including the presence of a pathogenic mutation in *TP53* to exclude low grade serous tumors. Our final dataset included samples subjected to the following tests: OmniSeq Comprehensive (OC, *n* = 64), OmniSeq Immune Report Card (IRC, *n* = 26), OminSeq Advance (OA, *n* = 97), and OmniSeq INSIGHT (OI, *n* = 219). The most common pathogenic alterations were observed in the following genes (Fig. 3): *BRCA1* (21.93%), *PIK3R1* (18.23%), *NF1* (17.98%), *APC* (15.76%), *ZFHX3* (15.02%), and *BRCA2* (14.53%). In lieu of an HRD score, we segregated cases from the OmniSeq cohort into three groups to evaluate the effect of HR gene alterations on molecular characteristics: (1) BRCAa, which included cases with alterations in *BRCA1* and/or *BRCA2* (*n* = 128, 31.5%); (2) NBHRa, which included cases with alterations in any non-*BRCA* HR-related genes (*n* = 114, 28.1%), and (3) HRi, defined as cases without alterations in HR-related genes (*n* = 164, 40.4%). Patients had a median age at test ordering of 65 years (range: 25–90). The BRCAa group contained younger patients than the NBHRa (median 65 vs. 71 years, *P* < 0.0001) and the HRi groups (65 vs. 70 years, *P* = 0.0003). There was no statistical difference between patient age in the NBHRa and HRi groups (*P* > 0.05).

A comparison of CGIP results from the three HR-related groups revealed significant differences across several variables (Supplemental Table 3). The mean TMB for all patients was 6.4 mut/Mb, with BRCAa patients showing a significantly higher mean TMB (12.1 ± 72.5) compared to either NBHRa (4.4 ± 2.48, *P* = 0.0052) or HRi (3.9 ± 2.3, *P* = 0.0059) groups, and the percentage of patients with high TMB (≥ 10 mutations/Mb) was notably higher in the BRCAa group (3.5%) compared to the HRi group (0.7%, *P* = 0.0043). Although PD-L1 IHC expression is not a predictive biomarker associated with sensitivity to FDA approved therapies for HGSOC patients, PD-L1 expression levels using TPS scoring criteria were available for all patients in this cohort. We observed no differences in PD-L1 TPS scores among the three groups (*P* > 0.05), and the proportion of PD-L1 positive patients (≥ 1% TPS) was similar across all groups, with 48.0% in BRCAa, 37.4% in NBHRa, and 44.1% in HRi, with no significant differences seen in pairwise comparisons. Tumor Immunogenicity Score (TIGS), a composite immune gene expression score, was significantly lower in the BRCAa group (40.8 ± 17.7) compared to the HRi group (46.5 ± 21.8, *P* = 0.033). BRCAa patients also had a lower frequency of strong TIGS (14.0%) compared to HRi (30.8%, *P* = 0.0022). CP expression score was significantly higher in the BRCAa group (51.8 ± 23.3) compared to the HRi group (44.4 ± 24, *P* = 0.036); however, no significant differences were observed across semi-quantitative CP score distributions (*P* > 0.05). CTAB expression score was higher for the HRi group (317.4 ± 236.5) compared to BRCAa tumors (285.2 ± 225, *P* = 0.014).

**Fig. 4:**
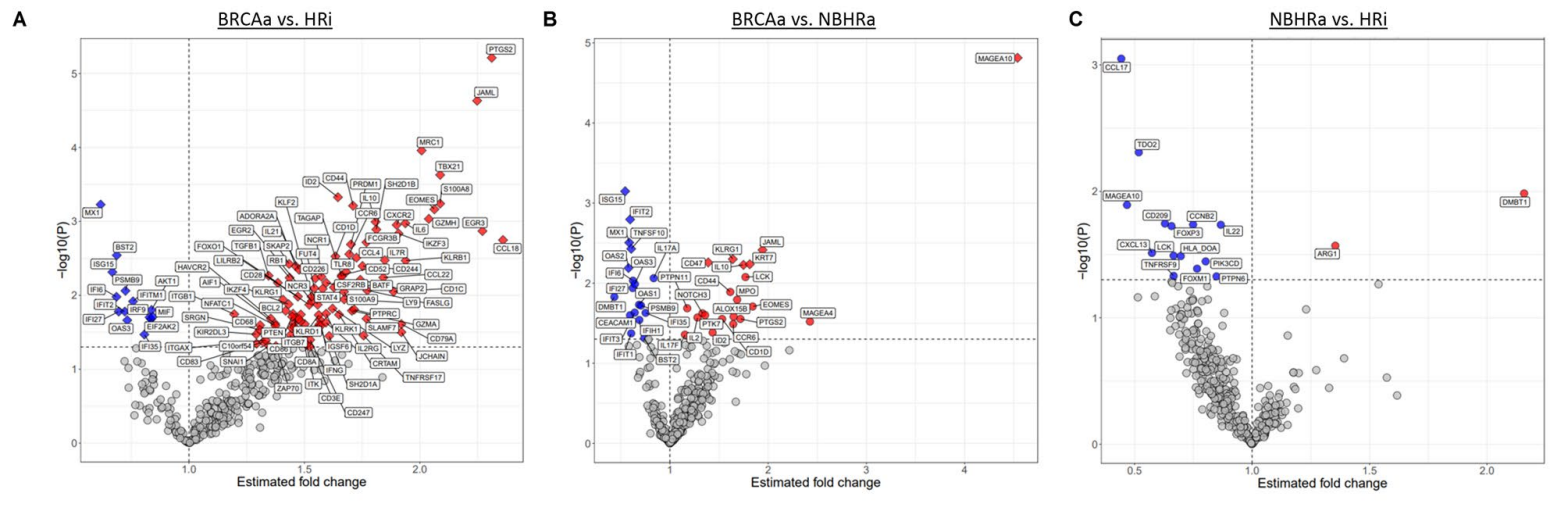
Gene expression differences observed between BRCAa, NBHRa, and HRi HGSOC. Volcano plots of statistical significance vs. fold change for HR-related groups from the OmniSeq HGSOC cohort. Diamond dots represent genes with FDR < 0.25 threshold, whereas circular dots represent genes with an uncorrected P-value < 0.05. (A) We observed 111 DEGs when comparing the BRCAa and HRi cohorts (blue = increased in BRCAa; red = increased in HRi). (B) We observed 11 DEGs when comparing gene expression of BRCAa to NBHRa HGSOC (blue = increased in BRCAa; red = increased in NBHRa). (C) We observed no statistically significant DEGs (i.e. FDR < 0.25) when comparing NBHRa to HRi tumors (blue = increased in NBHRa; red = increased in HRi).

Differential gene expression analysis revealed marked differences in gene expression between the BRCAa and HRi cohorts, with 14 DEGs elevated in BRCAa vs. 97 elevated in the HRi group (Fig. 4, Supplemental Table 4). The NBHRa group showed fewer gene expression changes, with 5 DEGs elevated in BRCAa vs. 6 in NBHRa and with no elevated DEGs with FDR < 0.25 when comparing the NBHRa and HRi groups.

### RB1 expression status is associated with distinct immune gene expression profiles

To evaluate the effect of RB1 mRNA expression on genomic features and immune profiles in the cohort, we stratified each HR-related groups based on *RB1* mRNA expression levels, designating the lower quartile as RB1 “low” and combining the upper three quartiles as RB1 “high”.

We identified 332 cases from the HGOSC cohort that completed gene expression profiling. The cohort was comprised of 114 (34.3%) RBL and 218 (65.7%) RBH malignancies, which included 44 (47.3%) RBL and 49 (52.7%) RBH BRCAa tumors, 28 (26.4%) RBL and 78% (73.6%) RBH NBHRa tumors, and 42 (31.6%) RBL and 91 (68.4%) RBH HRi tumors (Supplemental Table 5). Chi-Square analysis demonstrated that HRi (*P* = 0.0184) and NBHRa (*P* = 0.0030) cohorts were both enriched for RBH tumors vs. the BRCAa cohort. In general, RBL status was associated with lower TIGS (37.7 vs. 49.0, *P* < 0.0001), lower CP (44.3 vs. 50.9, *P* = 0.033), and reduced expression of BRCA2 (*P* < 0.0001), CD3 (*P* = 0.0002), CD20 (*P* < 0.0001), CD68 (*P* = 0.0012), and PTEN (*P* < 0.0001). No differences in Ki-67 mRNA expression were observed among groups in the cohort (*P* > 0.05). Within the BRCAa subgroup, RBL vs. RBH tumors showed no statistically significant differences in TIGS (38.9 vs. 42.6, *P* > 0.05); in contrast, RBH status was associated with higher TIGS in HRi tumors (36.7 vs. 51.0, *P* = 0.0009). Regarding individual immune-related gene differences, RB1-status in BRCAa tumors was not associated with differences in CD3, CD20, and CD68 expression (*P* > 0.05); in contrast, RBL tumors in the HRi cohort were associated with lower CD3 (*P* = 0.0078), CD20 (*P* = 0.0130), and CD68 (*P* = 0.045) mRNA expression.

**Fig. 5:**
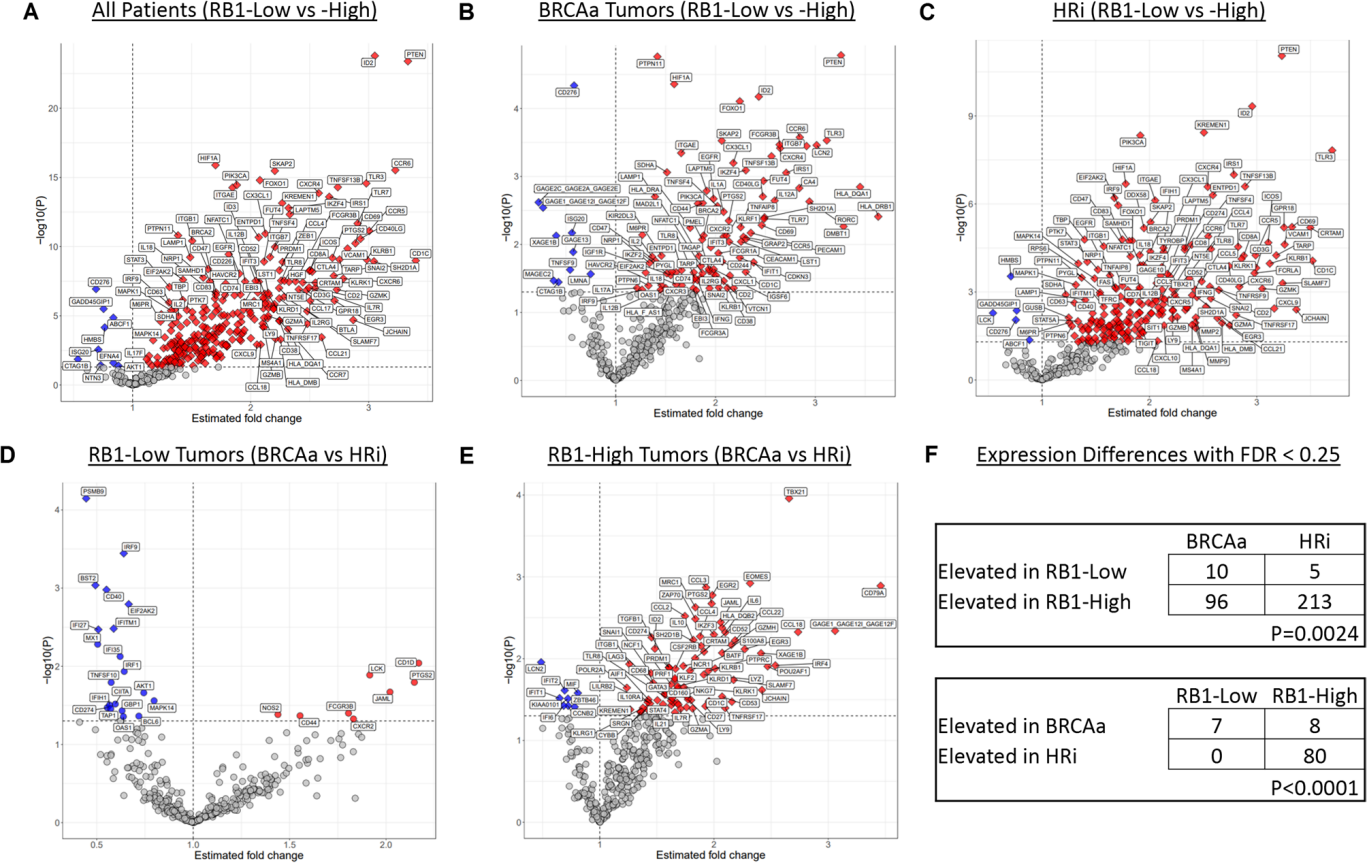
Gene expression changes associated with RB1 expression status and HR-related genes in HGSOC. Volcano plots of statistical significance vs. fold change for HR-related groups from the OmniSeq HGSOC cohort. Diamond dots represent statistically significant differentially expressed genes (DEGs, i.e. FDR < 0.25). Circular dots represent expression differences of potential interest with an uncorrected P-value < 0.05 and FDR > 0.25. (A) We identified 285 DEGs when comparing RBL to RBH tumors (blue = increased in RBL; red = increased in RBH). (B) There were 106 DEGs identified when comparing BRCAa-RBL (increased in blue) and BRCAa-RBH tumors (increased in red). (C) We identified 218 DEGs when comparing HRi-RBL (increased in blue dots) and HRi-RBH tumors (increased in red dots). (D) Examining RBL groups, we found 7 DEGs increased in BRCAa-RBL (blue diamonds) and no DEGs elevated in HRi-RBL tumors (red diamonds). (E) Among RBH groups, 8 DEGs were elevated in BRCAa-RBH tumors (blue) compared to 80 DEGs elevated in HRi-RBH tumors (red). (F) Chi-square analysis demonstrated that the proportion of DEGs was increased in RBH tumors (upper panel) and specifically in HRi-RBH tumors (lower panel).

Gene expression analysis revealed a smaller number of elevated DEGs in RBL vs. RBH tumors (9 vs. 276) in the OmniSeq cohort (Fig. 5, Supplemental Table 6). Among BRCAa tumors, RBL status was associated with 10 elevated DEGs in the BRCAa-RBL vs. 96 in the BRCAa-RBH samples. Among HRi tumors, RBL status revealed 5 DEGs with elevated fold change vs. 213 in the HRi-RBH group. Chi-square analysis demonstrated that the distribution of DEGs was different for BRCAa and HRi tumors when considering RB1 status, with a relatively greater proportion of DEGs occurring in the HRi-RBH vs. the BRCAa-RBH subgroup (*P* = 0.0024).

To further explore the differences in immune expression profiles, we compared molecular results between the HR-related subgroups restricted to either the RBL (Supplemental Table 7) or RBH subsets (Supplemental Table 8). Among RBL tumors, we observed a higher TMB (mut/Mb) in BRCAa vs. HRi tumors (5.2 vs. 3.9, *P* = 0.0058) and in BRCAa vs. NBHRa tumors (5.2 vs. 5, *P* = 0.039). In contrast, examination of the RBH tumors revealed that BRCAa tumors had lower TIGS than HRi tumors (42.6 vs. 51.0, *P* = 0.025) and lower CTAB than either HRi (232.6 vs. 327.6, *P* = 0.011) or NBHRa (232.6 vs. 362.8, *P* = 0.026) tumors. Among individual immune-related genes, CD68 mRNA expression was greater in HRi-RBH than BRCAa-RBH tumors (3266.4 vs. 2022.6 nRPM, *P* = 0.02).

Next, we evaluated fold changes in gene expression to compare the HR-related groups stratified by RB1 status (i.e., BRCAa-RBL vs. HRi-RBL and BRCAa-RBH vs. HRi-RBH). Among RBL tumors (Supplemental Table 9) BRCAa-RBL tumors exhibited 7 DEGs with elevated fold change compared to zero elevated DEGs in the HRi-RBL group. Among RBH tumors (Supplemental Table 10), BRCAa-RBH tumors had 8 DEGs with elevated fold change compared to 80 DEGs elevated in HRi-RBH tumors. Chi-square analysis showed that the proportion of elevated DEGs was significantly greater for HRi-RBH tumors (*P* > 0.0001).

### Pathway analysis of differentially-expressed immune-related genes

We performed pathway analysis of DEGs from the OmniSeq cohort using the PANTHER classification system to identify overrepresented Reactome pathways and calculate fold enrichment (FE) over expected values based on the reference gene list. DEG lists and pathways analysis results are detailed in Supplemental Table 11.

#### A. Stratification by HR status

Considering DEGs related to HR gene status, BRCAa tumors showed enrichment of pathways related to interferon signaling, including interferon α/β signaling (FE > 100, FDR = 7.72 × 10^−22^) and ISG15 antiviral mechanisms (FE = 62.1, FDR = 6.08 × 10^−3^). Conversely, HRi tumors exhibited overrepresentation of epithelial-mesenchymal transition (EMT; FE > 100, FDR = 0.0143), adaptive immune regulation (IL-4/IL-13 signaling; FE = 24.9, FDR = 2.21 × 10^−12^), and anti-inflammatory pathways including CD163 (FE = 70.7, FDR = 1.46 × 10^−3^) and IL-10 signaling (FE = 33, FDR = 5.32 × 10^−7^). Additionally, HRi tumors demonstrated DEGs of kinase pathways including PI3K/AKT signaling in cancer (FE = 11.5, FDR = 8.88 × 10^−3^), ALK (FE = 22.7, *P* = 0.0276), and NTRK1 (FE = 8.1, *P* = 0.0330).

#### B. Stratification by RB1 mRNA expression

Next, we stratified tumors according to RB1 mRNA expression status. We found no significantly overrepresented pathways in the RBL group, whereas the RBH group demonstrated enrichment of multiple signaling pathways: EMT (FE = 36.8, FDR = 0.0191), interferon α/β (FE = 14.5, FDR = 1.30 × 10^−11^), interferon γ (FE = 15.3, FDR = 4.12 × 10^−16^), chemokine receptor (FE = 33.5, FDR = 3.38 × 10^−31^), PD-1 (FE = 33.0, FDR = 3.79 × 10^−15^), and interleukin pathways (FE = 10.1, FDV = 5.82 × 10^−42^), including IL-4/13 (FE = 20.5, FDR = 1.11 × 10^−29^), IL-6 (FE = 12.3, FDR = 6.22 × 10^−3^), IL-10 (FE = 35.9, FDR = 3.87 × 10^−27^), IL-12 (FE = 11.8, FDR = 3.71 × 10^−6^), and IL-21 (FE = 44.1, FDR = 9.03 × 10^−8^). Additionally, kinase signaling pathways were represented in RBH tumors, such as PIK3K cascade (FE = 8.2, FDR = 0.0240), MET signaling of PI3K/AKT (FE = 29.4, FDR = 0.0299), and STAT3 nuclear events downstream of ALK signaling (FE = 33.4, FDR = 1.11 × 10^−5^).

#### C. Stratification of BRCAa tumors by RB1 status

Among BRCAa tumors, we identified no pathways enriched in RBL tumors compared to RB1 high tumors. However, BRCAa-RBH tumors showed enrichment in interferon α/β (FE = 19.8, FDR = 1.41 × 10^−5^), trafficking and processing of endosomal toll-like receptor (FE = 49.5, FDR = 1.93 × 10^−3^), chemokine receptor (FE = 30.1,FDR = 9.96 × 10^−8^), and interleukin signaling pathways, including IL-2 (FE = 35.7, FDR = 0.0481), IL-4/IL-13 (FE = 21.2, FDR = 2.13 × 10^−9^), IL-10 (FE = 33.4, FDR = 5.65 × 10^−7^), and IL-12 (FE = 15.3, FDR = 0.0074). Additionally, BRCAa-RBH tumors demonstrated DEGs of kinase pathways including PI3K/AKT (FE = 16.3, FDR = 4.92 × 10^−5^) and ALK (FE = 45.9, FDR = 9.98 × 10^−7^).

#### D. Stratification of HRi tumors by RB1 status

In an analysis restricted to HRi tumors alone, we identified no pathways enriched in HRi-RBL tumors over HRi-RBH tumors. However, HRi-RBH tumors showed enrichment of EMT (FE = 48.5, FDR = 0.0139) and interleukin-related signaling pathways, including IL-6 (FE = 35.3, FDR = 1.52 × 10^−4^), IL-9 (FE = 43.1, FDR = 6.80 × 10^−5^), IL-10 (FE = 41.0, FDR = 7.81 × 10^−24^), IL-12 (FE = 12.1, FDR = 8.30 × 10^−5^), and IL-21 (FE = 38.8, FDR = 1.02 × 10^−4^). Additionally, HRi-RBH tumors showed activation of the following kinase pathways over HRi-RBL tumors: PI3K/AKT (FE = 10.6, FDR = 2.70 × 10^−6^), ALK (FE = 15.3, FDR = 5.90 × 10^−11^), and MET (FE = 6.4, FDR = 0.0221).

#### E. Stratification of RBL and RBH tumors by HR status

Finally, we stratified tumors by RB1 expression status and compared DEGs elevated in BRCAa and HRi groups. Among RBL tumors, BRCAa was associated with interferon α/β signaling (FE > 100, FDR = 3.29 × 10^−8^), cytokine signaling (FE = 23.0, FDR = 1.57 × 10^−5^), and immune system activation (FE = 8.4, FDR = 4.61 × 10^−3^); HRi-RBL tumors showed no elevated differentially expressed genes with an FDR < 0.25 when compared to BRCAa-RBL tumors. Among RBH tumors, BRCAa was again associated with interferon α/β signaling genes (FE > 100, FDR = 7.00 × 10^−3^). Compared to BRCAa-RBH tumors, HRi-RBH tumors were associated with a variety of overrepresented pathways including EMT (FE > 100, FDR = 0.0179), IL-4/IL-13 (FE = 25.5, FDR = 2.27 × 1-^−10^), IL-10 (FE = 4.6, FDR = 2.61 × 10^−9^), and general receptor tyrosine kinase signaling (FE = 44.9, FDR = 0.0229).

## Discussion

**Fig. 6:**
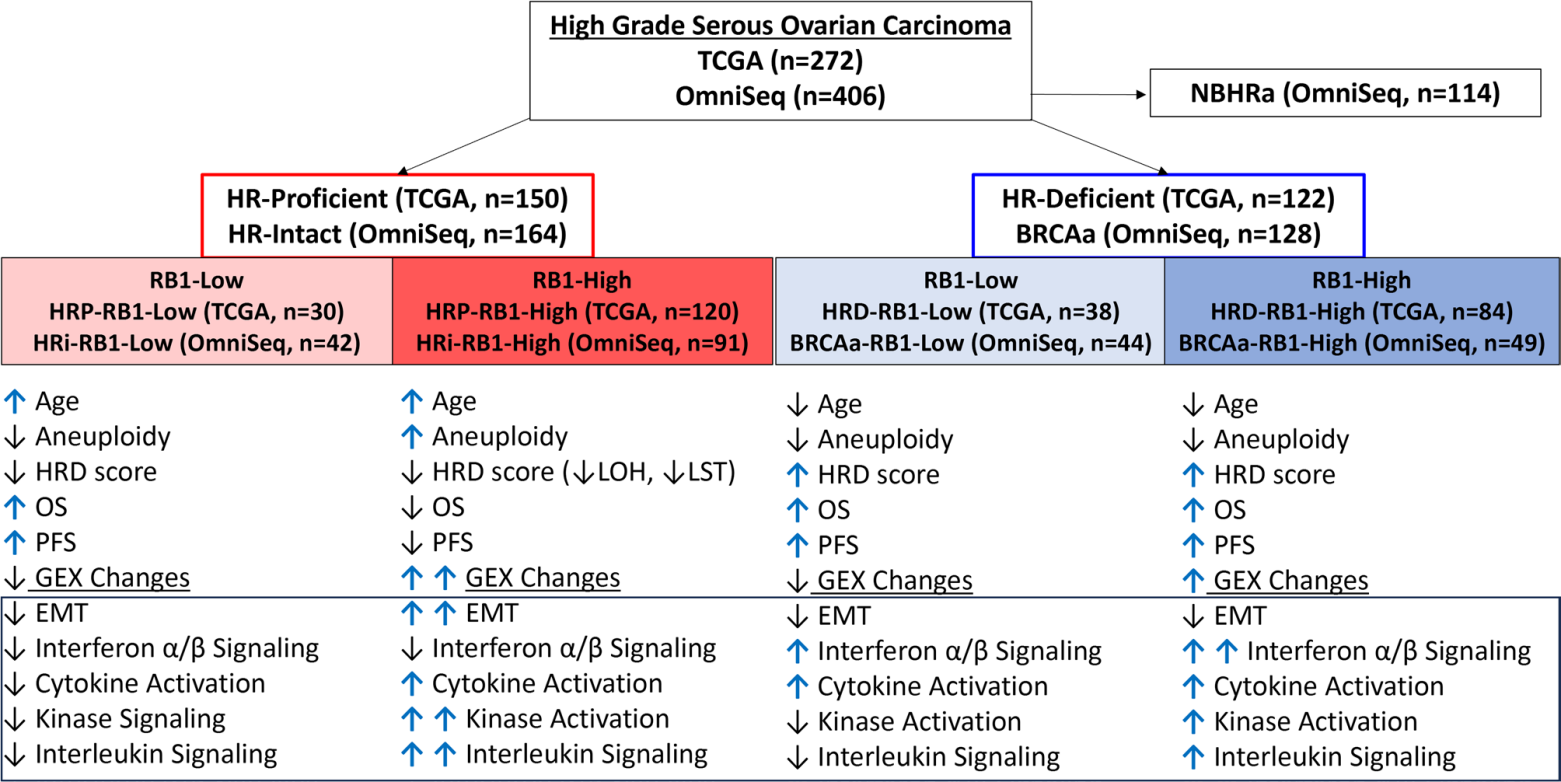
Summary of study cohorts and results. We examined two cohorts enriched for HGSOC, which were categorized based on HR scores (TCGA) or HR gene alterations (OmniSeq) and further subclassified based on RB1 mRNA expression status. As expected, HRD & BRCAa cohorts were associated with a lower age than HRP & HRi. OS and PFS was diminished in patients with HRP-RBH tumors, which were also associated with increased aneuploidy. Evaluation of immune-related gene expression was performed on tumors with intact HR genes, demonstrating that HRi-RBH tumors showed gene expression changes related to epithelial-mesenchymal transformation (EMT), potentially oncogenic kinase activation, and interleukin signaling. In contrast, BRCAa tumors showed relative activation of interferon α/β and cytokine activation relative to HRi tumors.

The aim of this study was to explore the relationship between HR status and RB1 mRNA expression in HGSOC. As summarized in Fig. 6, our results show that HRP-RBH tumors are a distinct molecular subgroup within HGSOC, characterized by aneuploidy, unique gene expression patterns, and poorer survival outcomes compared to other subgroups. These observations highlight the potential of HR and RB1 stratification to improve prognostic assessments and potentially tailor future therapeutic strategies.

The loss of RB1, commonly observed in HGSOC^4,9^, has long been implicated as a core driver of malignant transformation^12–15,38^. We confirmed that RB1 expression levels closely correlate with RB1 copy number, where homozygous deletions lead to the lowest RB1 mRNA expression, and amplifications result in the highest levels. This gene dosage effect aligns with prior studies^39,40^, highlighting RB1 deletion as a major mechanism for its loss of function. However, in tumors with hemizygous deletions or low RB1 expression, epigenetic mechanisms, such as promoter hypermethylation, may silence the remaining allele, contributing to RB1 loss as seen in other tumor types^41–45^. Moreover, our analysis identified several inactivating single nucleotide variants (SNVs) in RB1, which, although infrequent, may substantially impair its function. These variants typically involved frameshift or splice site mutations, aligning with the role of RB1 as a critical protein-binding cell cycle checkpoint protein^40^. Notably, we also observed concordance between RB1 mRNA and protein levels within the TCGA cohort, supporting the use of mRNA as a surrogate marker for protein expression. Future studies incorporating immunohistochemistry or proteomic profiling may help validate the mRNA cutoffs used and potentially enable RB1 stratification for clinical purposes.

RBH tumors, particularly within the HRi group, showed elevated DEGs of immune signaling pathways (e.g., IL-10, IL-4/13) and oncogenic kinase signaling pathways (e.g., PI3K/AKT, MET). These pathways suggest that RBH tumors are not only driven by tumor-protective immune regulation but also by pro-survival signaling. In contrast, RBL tumors lacked DEGs of specific pathways. Of note is that patients with HRP-RBH tumors in the TCGA cohort demonstrated the poorest survival metrics across all subgroups, but the mechanisms driving poor survival in this context are uncertain and should be investigated further. Future studies should investigate the relationship between RB1 expression and platinum resistance.

RB1-status in HGSOC may have therapeutic implications because inhibition of CDK4/6 has been proposed as a therapeutic target^9,46,47^. In normal cell cycle regulation, phosphorylation of RB1 by CDK4/6 results in its disassociation from E2F1/2/3, resulting in transcriptional activation. Thus, inhibition of these RB1 regulators prevents inhibitory phosphorylation allowing RB1 to inhibit cellular proliferation and delay disease progression^48–50^. However, there is strong evidence that loss of RB1 removes the evolutionary advantage provided by CDK4/6 deregulation^51^; in other words, CDK4/6 inhibitors may not have any effect in tumors with loss of RB1 expression. Therefore, RB1 loss should be considered in exclusion criteria for trials that target CDK4/6 activity.

Our study is orthogonal and complementary to existing molecular classifications of HGSOC^4,52,53^, notably the four major transcriptomic subtypes (immunoreactive, differentiated, proliferative, mesenchymal), by evaluating just two variables: HR status and RB1 expression. BRCAa tumors, resembling the immunoreactive subtype, exhibited a favorable prognosis and enhanced activation of innate immune responses, particularly through interferon signaling. This immune activation is likely driven by genomic instability, leading to the accumulation of neoantigens and cytosolic DNA^54–56^. In contrast, HRi-RBH tumors shared similarities with the mesenchymal subtype, demonstrating upregulation of immune evasion mechanisms, including anti-inflammatory pathways (e.g., IL-10, CD163) and EMT, suggesting these tumors rely on these mechanisms to promote tumor progression and evade immune detection. Of note, we observed a lack of significant gene expression changes in the NBHRa subgroup, suggesting that NBHRa included both HR-deficient and HR-proficient tumors, supporting its exclusion from our downstream analyses.

The current study has several important limitations. As a retrospective study, it is inherently limited by the quality and completeness of the available data. While HR status was available for the TCGA cohort, we had to rely on gene alterations to assess HR status in the OmniSeq cohort, which may not be as reliable. The potential misclassification of HR status based on gene alterations alone may not capture the full complexity of gene expression changes associated with HR proficiency, leading to possible inaccuracies. Furthermore, our study’s findings are based on a cohort of advanced HGSOC undergoing CGIP, and external validation is needed to ensure generalizability. Future studies should aim to replicate our findings in larger, more diverse datasets with well-defined HR status for each case.

In conclusion, our study provides evidence for the molecular subtyping of HGSOC based on HR status and RB1 expression. We identified HRP-RBH tumors as the largest subgroup, characterized by the poorest prognosis and a distinct genomic instability profile marked by high aneuploidy and particularly low levels of HR deficiency. Molecular stratification of HGSOC based on HR status and RB1 expression may have implications for prognosis and therapeutic decision-making, with the potential to identify patients with platinum-resistant tumors. Our findings highlight the potential of RB1 mRNA expression as a prognostic biomarker, particularly for patients with HRP HGSOC. Given the distinct molecular, immune and prognostic profiles of HRP-RBH tumors, these patients may benefit from alternative follow-up strategies. CDK4/6 inhibitors, which target cell cycle regulation, could be promising for RB1-intact HGSOC, although further studies are needed to confirm their efficacy, particularly considering resistance mechanisms associated with RB1 loss.

## Supplementary Information

Below is the link to the electronic supplementary material.

Supplementary Material 1

Supplementary Material 2

Supplementary Material 3

Supplementary Material 4

Supplementary Material 5

Supplementary Material 6

Supplementary Material 7

Supplementary Material 8

Supplementary Material 9

Supplementary Material 10

Supplementary Material 11

## Data Availability

Data from the TCGA cohort is publicly accessible through The Cancer Genome Atlas repository (http://cancergenome.nih.gov). Clinical data from the OmniSeq cohort are not publicly available due to patient confidentiality and data-sharing agreements. Researchers may contact the corresponding author for inquiries regarding potential collaboration and data sharing.
