# Correction: RB1 expression and HR proficiency define a poor prognosis subtype of high grade serous ovarian cancer

**DOI:** 10.1038/s41598-025-23530-w

**Published:** 2025-10-23

**Authors:** Kyle C. Strickland, Zachary D. Wallen, Heidi C. Ko, Michelle F. Green, Alicia Dillard, Sarabjot Pabla, Stephanie Hastings, Alison Roos, Taylor J. Jensen, Marcia Eisenberg, Brian J. Caveney, Shakti Ramkissoon, Eric A. Severson, Rebecca A. Previs

**Affiliations:** 1 Labcorp, 10 Moore Dr, Durham, NC 27703 USA; 2 Department of Pathology, Duke University Medical Center, Duke Cancer Institute, Durham, NC 27710 USA; 3 Thermo Fisher Scientific, Carlsbad, CA 92008 USA; 4 Labcorp, Burlington, NC 27215 USA; 5 Department of Pathology, Wake Forest Comprehensive Cancer Center, Wake Forest School of Medicine, Winston-Salem, NC 27109 USA; 6 Department of Obstetrics & Gynecology, Division of Gynecologic Oncology, Duke University Medical Center, Duke Cancer Institute, Durham, NC 27710 USA

Correction to: *Scientific Reports* 10.1038/s41598-025-15156-9, published online 12 August 2025

The original version of this Article contained an error where an incorrect version of Fig. 3 was published. The original Fig. 3 and accompanying legend appear below.

**Fig. 3:**
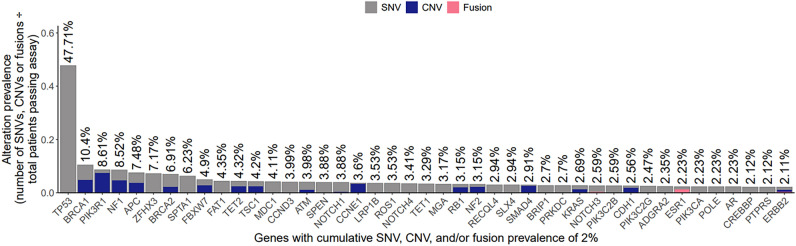
Genomic landscape of cases from the OmniSeq HGSOC cohort. The histogram below shows the prevalence and type of alterations for the most commonly altered genes in the OmniSeq HGSOC cohort. Of note, 100% of the tumors harbored pathogenic alterations in *TP53* (not shown).

The original Article has been corrected.

